# Fabrication of Crosslinked Poly(L-lactic acid) with Enhanced Shape Memory Performance via γ-Ray Irradiation

**DOI:** 10.3390/polym17223041

**Published:** 2025-11-17

**Authors:** Jiayao Wang, Jingxin Zhao, Dong Yang, Jichun You, Guipeng Yu

**Affiliations:** 1 College of Chemistry and Chemical Engineering, Central South University, Changsha 410083, China; 2 College of Material, Chemistry and Chemical Engineering, Key Laboratory of Organosilicon Chemistry and Material Technology, Ministry of Education, Zhejiang Key Laboratory of Organosilicon Material Technology, Hangzhou Normal University, Hangzhou 311121, China; 3 Department of Cardiovascular Medicine, Affiliated Hospital of Hangzhou Normal University, Hangzhou 310015, China

**Keywords:** PLLA, shape memory, crosslinking, γ-ray irradiation

## Abstract

In this work, an innovative approach has been established to overcome the intrinsic limitations of poly(L-lactic acid) (PLLA)-based shape memory polymers (SMPs) by introducing γ-ray-induced crosslinking in miscible PLLA/triallyl isocyanurate (TAIC) blends. The crosslinking density can be precisely adjusted by the TAlC content and absorbed irradiation dose, enabling the fabrication of PLLA SMPS with outstanding shape memory properties. In crosslinked PLLA SMPs, the crosslinking point can significantly suppress the cold crystallization and prevent the irreversible chain slippage during deformation in the shape memory process, resulting in not only a high shape recovery ratio (99.5% at 10 wt% TAIC) but also a good cycle stability (maintaining 97.9% after three cycles). Moreover, the crosslinking points as well as the PLLA crystals endow PLLA with triple-shape memory performance, in which both the glass transition temperature and melting temperature serve as switch conditions. Remarkably, even under extreme deformation conditions (strain up to 800%), the crosslinked PLLA SMPs maintain a recovery ratio as high as 99.3%. Our results offer a novel strategy for fabricating high-performance PLLA SMPs with exceptional shape recovery capabilities.

## 1. Introduction

Thermal shape memory polymers (TSMPs), as a type of temperature-responsive material, have attracted significant attention due to their unique capability to be programmed into a temporary shape and subsequently return to their permanent shape upon exposure to a specific thermal stimulus [1,2,3,4,5,6,7,8]. The shape memory performance of TSMPs is fundamentally determined by the synergistic interplay between their soft and hard segments. The soft segments undergo reversible phase transitions between a flexible, rubbery state and a rigid, glassy state, which enables the fixation of temporary shapes. In contrast, the hard segments provide permanent network stability and support reversible deformation by minimizing chain slippage. Owing to these characteristics, TSMPs exhibit significant potential for application across a diverse range of fields, such as biomedicine [9,10,11], smart textiles [12,13], and 4D printing [14,15,16].

Poly(lactic acid) (PLA), a semicrystalline polymer with shape memory properties, biocompatibility, biodegradability, and environmental friendliness, has received increasing attention in the development of TSMPs [17,18,19]. PLA can be synthesized from both L- and D-lactic acid, with poly(L-lactic acid) (PLLA) being the L-isomer and poly(D-lactic acid) (PDLA) the D-isomer [20]. PLLA is commonly used, whereas PDLA, due to its chiral nature, is often employed in the formation of stereocomplexes with PLLA, which enhances the mechanical properties, crystallinity, and thermal stability of the resulting material [21]. As for the shape memory performance of PLLA, the amorphous regions function as reversible switching segments based on the glass transition temperature (*T*_g_), whereas the crystalline regions in PLLA act as physical crosslinking points that stabilize the permanent shape. However, conventional linear PLA exhibits poor shape recovery ratios under conditions of large deformation, primarily due to chain slippage and strain-induced crystallization above *T*_g_, thereby limiting its broader application [22,23,24]. Hence, many efforts have been made to improve the shape memory performance of PLA, including blending [25,26,27,28,29] and copolymerization [30,31,32]. Among these, blending PLA with flexible polymers or functional fillers has emerged as a particularly effective method for enhancing its toughness and ductility, which in turn improves both shape fixity and shape recovery performance [33]. For instance, the addition of nanofillers such as graphene oxide (GO), cellulose nanocrystals (CNCs), and cellulose ester (CE) not only reinforces the mechanical properties of the PLA matrix but also accelerates thermal responsiveness by increasing crystallinity and improving thermal conductivity [34]. However, a major challenge in blending strategies is the weak interfacial interactions between PLA and other components. These incompatibilities can cause structural defects that lead to stress concentration during deformation, ultimately reducing mechanical performance [35,36]. Alternatively, copolymerization is a molecular-level approach to tailoring the shape memory properties of PLLA. The introduction of flexible segments or functional comonomers in the chain of PLLA can reduce chain rigidity, adjust thermal transition temperatures, and create reversible phases that facilitate shape fixation and recovery. For instance, Fan et al. synthesized a series of amorphous PLA-based poly(ester urethane) elastomers using different PLA stereoisomers. The combination of a unique molecular architecture, enhanced chain entanglements, and hydrogen bonding interactions between urethane groups endows the PLDU elastomers with an excellent one-way shape memory effect, achieving nearly 100% shape fixity and recovery [37]. However, copolymerization strategy is often complex and costly and the improvements in shape memory performance are still limited. So, enhancing the shape memory performance of PLA remains a key challenge and research focus in this field.

**Figure 1:**
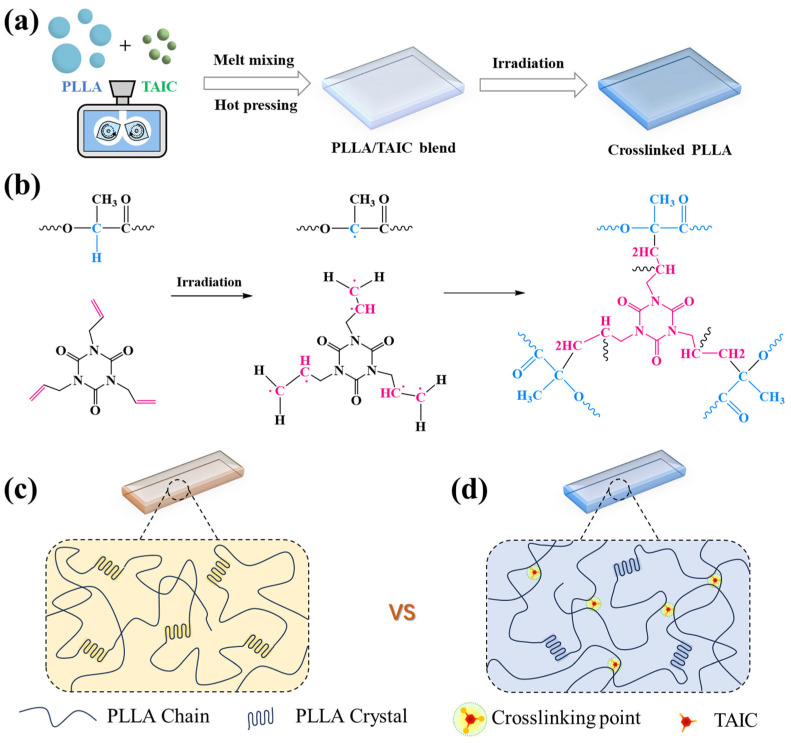
Schematic illustrations of (**a**) materials processing, (**b**) γ-irradiation-induced crosslinking mechanism, (**c**) conventional PLLA SMPs only with crystals, and (**d**) SMPs with crystals and crosslinking points.

In this work, the irradiation crosslinking method has been employed to tailor the shape memory properties of PLA through precisely controlled crosslinking by taking PLLA/TAIC (Triallyl isocyanurate) as an example (Figure 1a–d). TAIC is one of the most effective crosslinking agents for PLA. Monomer radicals can be created upon irradiation due to the breaking of the double bonds of the TAIC, which subsequently react with the radicals on PLLA to form crosslinking points [38,39]. Our previous work has demonstrated that TAlC exhibits excellent miscibility with PLLA [40]. Upon γ-ray irradiation, TAlC serves as an efficient crosslinker to form covalent connections between PLLA chains [41]. For one thing, the crosslinking density can be adjusted by varying the TAIC content and irradiation dose. For another, the crosslinking points can effectively restrict the chain slippage, which is beneficial for improving both the recovery ratio and fixation efficiency under large deformations. In the end, the PLLA/TAIC composites, containing two kinds of hard segments (tiny crystals and crosslinking points), turn into tripe TSMPs. Compared to traditional methods, such as blending and copolymerization, the irradiation crosslinking method offers a simple, efficient, and solvent-free strategy to overcome the inherent limitations of PLA for high-performance biodegradable shape memory applications.

## 2. Materials and Methods

### 2.1. Materials and Sample Preparation

PLLA (3001D) with a weight-average molecular weight (*M*_w_) of 158,000 g/mol was supplied by Nature Works (Blair, NE, USA). Triallyl isocyanate (TAIC) was provided by Sinopharm Chemical Reagent Co., Ltd. (Shanghai, China). Prior to melt blending, PLLA was dried in a vacuum oven at 80 °C for 12 h. Subsequently, PLLA and TAIC were premixed in a Haake mixer (Thermo Fisher Scientific, Haake Polylab QS, Waltham, MA, USA) at 190 °C with a rotation speed of 50 rpm for 10 min to form a homogeneous PLLA/TAIC blend. The TAIC weight fractions in the blend are set to 1%, 3%, 5%, and 10%. The blends were hot-pressed at 190 °C under a pressure of 10 MPa to obtain a film with a thickness of 300 μm. The specimens were vacuum sealed and then irradiated by γ-ray from a ^60^Co source with a dose of 30 kGy at room temperature (Figure 1a).

### 2.2. Characterizations

The morphologies of PLLA/TAIC blends were investigated by means of a Field Emission Scanning Electron Microscope (FESEM, Hitachi, S-4800, Tokyo, Japan) with an accelerating voltage of 5 kV. The crystallization and melting behavior of the PLLA/TAIC blends before and after crosslinking were also investigated with the help of differential scanning calorimetry (DSC, TA, Q2000, New Castle, DE, USA) under a dry nitrogen flow. The specimens were heated from 0 °C to 200 °C and held at 200 °C for 5 min to erase the thermal history. Then the specimens were cooled from 200 °C to 0 °C. The heating and cooling rates were 10 °C/min. The crystallinity (Xc) can be calculated by the following Equation (1):

(1) $$X_{c}=\frac{∆H_{m}-∆H_{cc}}{{∆H}_{m}^{o}}\times 100\%$$

where $∆H_{m}$, $∆H_{cc}$, and ${∆H}_{m}^{o}$ are actual melting enthalpy, cold crystallization enthalpy, and standard melting enthalpy (93 J/g) for PLLA, respectively [42].

Fourier transform infrared (FT-IR) spectra of PLLA, TAIC, and crosslinked PLLA were recorded using a Bruker spectrometer (Bruker, VERTEX 70v, Ettlingen, Germany), with a resolution of 4 cm^−1^ and an accumulation of 64 scans, covering the mid-IR region from 4000 to 500 cm^−1^. The glass transition temperature (*T*_g_) of crosslinked PLLA/TAIC specimens was measured by dynamic thermomechanical analysis (DMA, TA, Q800, Wilmington, DE, USA) at an oscillation frequency of 5 Hz. The gel weight of the crosslinked PLLA/TAIC specimen can be calculated based on Equation (2).

(2) $$Gel\;weight=\frac{m_{48}-m_{0}}{m_{0}}\times 100\%$$

where $m_{0}$ and $m_{48}$ are the weights of the dried specimen and the chloroform-swelled specimen.

The mechanical behavior of crosslinked PLLA specimens at 80 °C was investigated with the help of an Instron Universal Testing Machine (Instron, Model 5966, Natic, MA, USA) at a speed of 10 mm/min.

### 2.3. Shape Memory Polymer Evaluation

The shape memory performance was measured by a uniaxial stretching experiment. The neat PLLA film and crosslinked PLLA were cut into a rectangular shape (~15 mm × 3 mm × 0.3 mm). For dual-shape memory performance, the specimens were stretched until the strain reached 100% in hot water (80 °C), followed by rapid cooling in cool water (25 °C) for 10 s to fix the temporary shape. Then, the deformed specimens were put into the hot water to recover their shape. The shape fixation ratio (*R_f_*) and shape recovery ratio (*R_r_*) were calculated by following Equations (3) and (4):

(3) $$R_{f}=\frac{L_{1}^{\prime }-L_{0}}{L_{1}-L_{0}}\times 100\%$$

(4) $$R_{r}=\frac{L_{1}^{\prime }-L_{0}^{\prime }}{L_{1}^{\prime }-L_{0}}\times 100\%$$

where *L*_0_ is the original length, *L*_1_ is the stretched length, *L*_1_′ is the fixed length, and *L*_0_′ is the recovery length.

To evaluate triple-shape memory performance, specimens were first stretched to approximately 100% strain at 170 °C in methyl silicone oil and subsequently fixed at 80 °C in water. The fixed strips were then stretched to approximately 200% of their original length at 80 °C in water and finally fixed at 25 °C in cooled water. Finally, the specimens first underwent shape recovery in water at 80 °C, followed by recovery in methyl silicone oil at 170 °C. All measurements were repeated at least three times. The ultimate recoverable deformation of PLLA SMPs with crystals and crosslinking points was evaluated by stretching specimens to 800% elongation at 80 °C, followed by recovery first at 80 °C and then at 170 °C. The shape fixation ratio (*R_f_*) and shape recovery ratio (*R_r_*) of triple-shape memory were also calculated based on Equations (3) and (4).

## 3. Results

**Figure 2:**
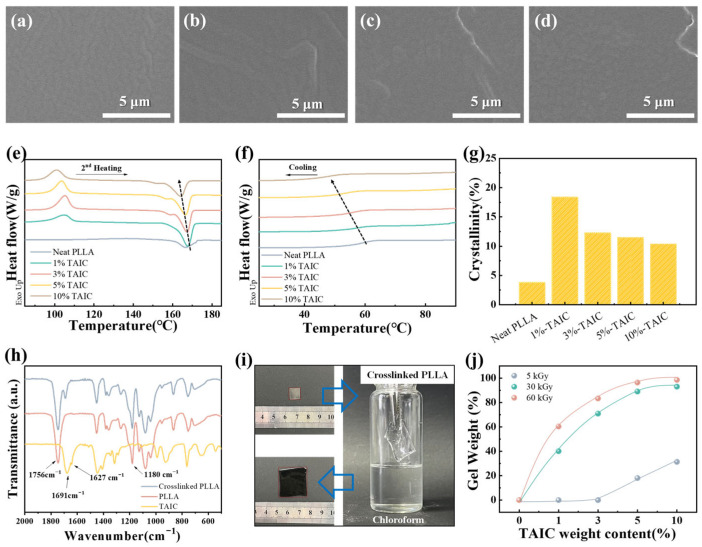
SEM images of the neat PLLA (**a**) and the PLLA/TAIC blends with 1 wt% (**b**), 3 wt% (**c**), and 10 wt% (**d**); TAIC before irradiation; the DCS curves of the PLLA/TAIC blend with different compositions (**e**,**f**) before irradiation; the crystallinities of neat PVDF and PLLA/TAIC blend with various content of TAIC before irradiation (**g**); the FT-IR spectra of PLLA, TAIC, and crosslinked PLLA (**h**); the solubilities of crosslinked PLLA ((**i**), 3 wt%-30 kGy) in chloroform (**i**); the gel weight of PLLA/TAIC blend with different compositions after irradiation at various doses (**j**).

The miscibility of PLLA and TAIC was initially investigated using SEM. As shown in Figure 2a–d, the cross sections of PLLA/TAIC blends exhibit homogeneity with no TAIC aggregation, even at TAIC contents up to 10 wt%. The DSC results are shown in Figure 2e,f. Firstly, the addition of TAIC significantly promotes the cold crystallization of PLLA during the heating process. Secondly, the melting temperature (*T*_m_) of PLLA decreases from 167.2 °C to 164.1 °C with increasing TAIC content up to 10 wt%, which is consistent with the melting point depression effect reported by Nishi and Wang [43]. Concomitantly, the glass transition temperature (*T*_g_) of PLLA declines significantly from 59.0 °C to 47.6 °C upon TAIC incorporation. These results confirm that TAIC exhibits excellent miscibility with PLLA, indicating its potential for forming crosslinking points in PLLA. The crystallinities of PLLA in the blends are 18.4%, 12.3%, 11.5%, and 10.4% when TAIC content increases from 1 wt% to 10 wt% (Figure 2g). The proposed crosslinking reaction mechanism during γ-ray irradiation is illustrated in Figure 1b. As reported by Quynh [38,44] and Yang [39], the allyl double bonds in TAIC break to produce active radicals that react with macroradicals on the PLA backbone through hydrogen abstraction, resulting in a three-dimensional crosslinked structure. Similar radical mechanisms were confirmed in subsequent studies on γ-irradiated PLA/TAIC blends [41]. As shown in Figure 2h, FT-IR was used to investigate the crosslinking reaction in the PLLA/TAIC blend. The peak at 1630 cm^−1^, corresponding to the C=C of TAIC, disappeared, indicating that the carbon–carbon double bonds have reacted with the radicals on PLLA during γ-ray irradiation. Furthermore, the presence of the characteristic peak at 1691 cm^−1^ (carbonyl on TAIC) confirmed its incorporation into the crosslinked network. After irradiation, the gel weight of the crosslinked PLLA was evaluated by the solvent swelling method. Due to the crosslinking points in crosslinked PLLA, the crosslinked PLLA exhibits a significant swelling phenomenon (Figure 2i). The gel weight increases with the increasing content of TAIC and irradiation dose, indicating the enhancement of the crosslinking density (Figure 2j), which is consistent with the results reported by Kodal [41]. These results demonstrate that the crosslinking density is controllable through TAIC concentration and irradiation dose, with 30 kGy determined as the optimal dose for achieving accurate regulation of crosslinking density.

**Figure 3:**
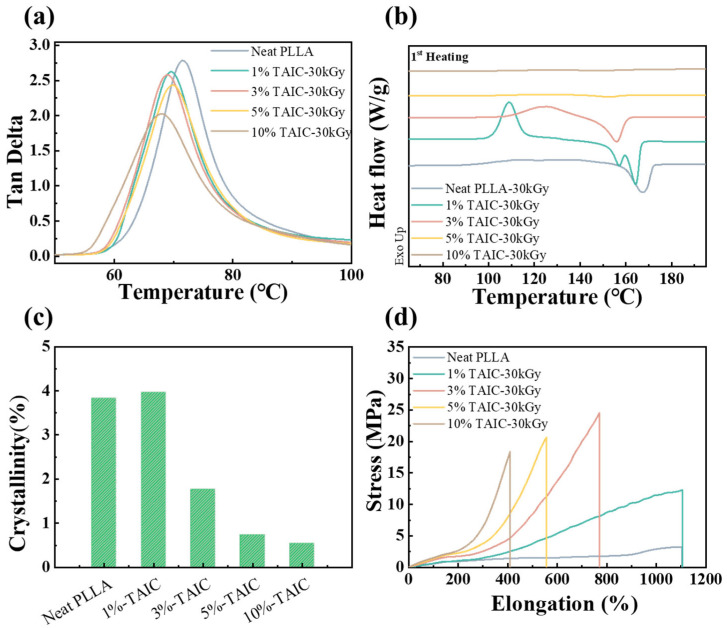
The glass transition of PLLA SMPs (**a**); the DSC curves (**b**), crystallinity (**c**), and stress–strain curves (**d**) of PLLA SMPs with various crosslinking densities.

It is essential to determine the glass transition temperature (*T*_g_) to serve as the switch condition between the rubber state and glass state. In Figure 3a, the glass transition temperature (*T*_g_) of neat PLLA is 71.4 °C. After irradiation, the *T*_g_ decreases from 69.6 °C to 67.9 °C as the TAIC content increases from 1 wt% to 10 wt%. This reduction in *T*_g_ can be attributed to an increase in free volume, resulting from the restricted packing of polymer chains due to crosslinking. The shape memory performance is well correlated with the crystals in PLLA. The high crystallinity and continuous crystal framework will influence the shape recovery ratio. For this purpose, the melting behavior of crosslinked PLLA has been investigated with the help of DSC. As shown in Figure 3c, the crystallinities of crosslinked PLLA with 0 wt%, 1 wt%, 3 wt%, 5 wt%, and 10 wt% TAIC are 3.8%, 4.1%, 2.7%, 1.2%, and 0.8%, indicating an obviously negative correlation between the crystallinity and gel weight of crosslinked PLLA. The swelling test results demonstrated that increasing TAIC concentration and irradiation dose lead to higher gel content, thereby confirming an enhanced crosslinking density, which accounts for the observed reduction in crystallinity. The results above suggest that the introduction of crosslinking points effectively hinders chain mobility, thereby suppressing the crystallization of PLLA. The mechanical properties of SMPs play an essential role in the shape memory behavior. The stress–strain curves of PLLA SMPs at 80 °C are illustrated in Figure 3d. Due to the rubbery state of PLLA above *T*_g_, the neat PLLA exhibits a high elongation at break (1104%) and low tensile strength (3.2 MPa). With increasing TAIC content, the tensile strength of PLLA SMPs increases from 3.2 MPa to 18.1 MPa due to the enhanced crosslinking density, while the elongation at break concurrently declines from 1104% to 432% (Table S1). Meanwhile, the elastic modulus of PLLA SMPs increases from 775 KPa to 1974 KPa as the crosslinking density increases, despite the decrease in crystallinity caused by crosslinking (Figure S1). The results above demonstrate that the crosslinking network is an ideal candidate to improve the shape memory performance and meet its requirements. The thermal stability of PLLA before and after irradiation has been investigated with the help of TGA. Taking PLLA with 3 wt% TAIC as an example, the thermal decomposition temperatures of starting (5% loss, *T*_5%_) and maximum (*T*_max_) for the PLLA were investigated and shown in Figure S2. The *T*_5%_ of neat PLLA before irradiation, neat PLLA after irradiation, and PLLA with 3 wt% TAIC before irradiation and after irradiation are 322.0 °C, 325.3 °C, 317.6 °C, and 328.7 °C, respectively. Their *T*_max_ values are 359.5 °C, 358.5 °C, 360.1 °C, and 362.5 °C. It can be concluded that the formation of crosslinking points significantly enhanced the thermal stability of PLLA.

**Figure 4:**
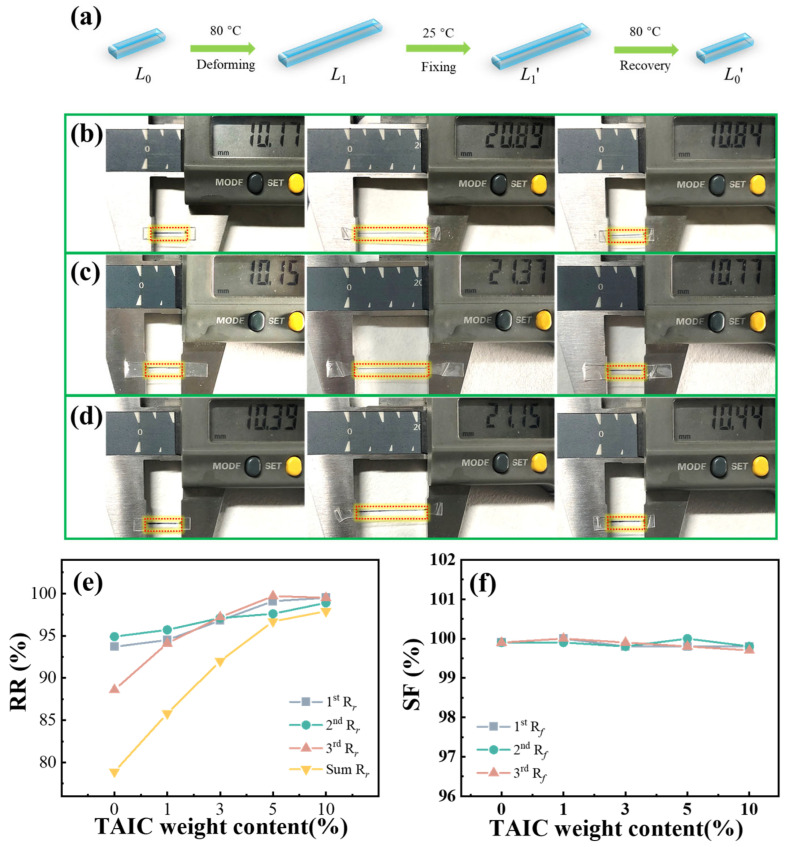
The tensile mode for evaluating the recovery and fixation ratio of PLLA SMPs (**a**); photos of one shape memory process of pure PLLA (**b**) and crosslinked PLLA (**c**) 1 wt%-30 kGy (**d**) 10 wt%-30 kGy; the shape recovery ratios of PLLA SMPs in three cycles (**e**); the shape fixity ratios of PLLA SMPs in three cycles (**f**).

The shape memory effects of PLLA SMPs were evaluated in the tensile mode and the results are shown in Figure 4a–f. The PLLA SMPs were initially stretched at 80 °C and were then fixed in the temporary shape at 25 °C. Finally, the PLLA SMPs were placed at 80 °C for shape recovery. In the first deformation and recovery cycle, Figure 4e shows that the shape recovery ratios increase from 93.8% to 94.5%, 96.8%, 99.1%, and 99.5% as the content of TAIC increases, indicating the positive impact of the crosslinking point on the shape recovery performance. Meanwhile, the shape fixation ratios in all specimens are over 99.5% (Figure 4f). In the second cycle, the PLLA SMPs exhibit similar shape recovery ratios and shape fixed ratios as in the first cycle. Nevertheless, after three cycles, the shape recovery ratio of PLLA SMPs without crosslinking points dropped to 78.9%, whereas that of PLLA SMPs with 10 wt% TAIC remained at 97.9%. It can be interpreted that the crosslinking points in PLLA significantly suppress the cold crystallization of PLLA above 80 °C. On the contrary, in the PLLA without crosslinking points, the rapid cold crystallization during the stretching process above *T*_g_ accounts for the increasing crystallinity and sharply declining recovery ratio. Therefore, the introduction of crosslinking points into the PLLA can efficiently weaken the cold crystallization and result in a high shape recovery ratio and excellent cyclic stability.

**Figure 5:**
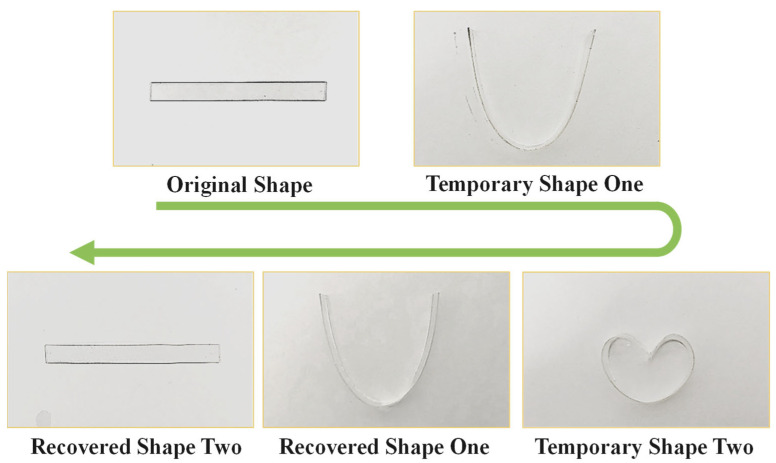
The triple-shape memory experiment of PLLA SMPs (3 wt% TAIC, 30 kGy) in the blending model.

The bending model was used to evaluate the triple-shape memory performance of PLLA SMPs. Taking PLLA SMPs with 3 wt% as an example, as depicted in Figure 5, the specimen was sequentially programmed by first melting at 170 °C, deforming into a U-configuration, and fixing as the temporary shape one at 80 °C. Due to the crosslinking points in PLLA, the crosslinked PLLA is still self-supporting at 170 °C. Subsequently, the specimen was further folded into a heart-like shape as the temporary shape two at 80 °C and fixed at 25 °C. During recovery, the heart-like shape specimen reverted to a U-like shape at 80 °C, following recovery to straight at 170 °C in methyl silicone oil. The result above demonstrates that the crosslinking point and crystal cooperatively establish two fixation points, thereby enabling triple-shape memory capability.

**Figure 6:**
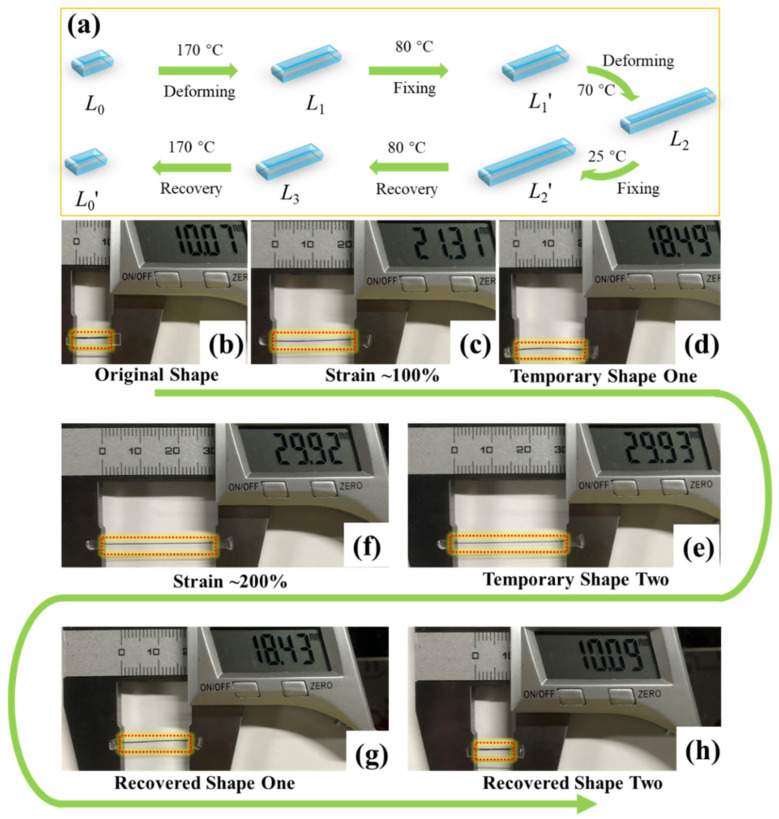
The tensile mode for evaluating the recovery and fixation ratio of PLLA SMPs with triple-shape memory (**a**); the shape memory process of PLLA SMPs (3 wt% TAIC, 30 kGy) (**b**–**h**).

The quantitative determination of triple-shape memory performance was also evaluated in the stretching model and the photos of PLLA SMPs (3 wt% TAIC, 30 kGy) during the shape memory process are shown in Figure 6a. The specimen was initially stretched to 100% strain at 170 °C and fixed at 80 °C, achieving the first temporary shape. It was then stretched further to 200% at 80 °C and fixed at 25 °C, establishing the second temporary shape. During recovery, the specimen underwent stepwise recovery at 80 °C and 170 °C. The shape fixation ratio ($R_{f}$) and shape recovery ratio ($R_{r}$) were calculated and are shown in Table 1. With the enhancement of crosslinking density, the $R_{f}^{1}$ significantly decreased from 99.9% to 74.9%, 52.7%, and 42.2%, whereas $R_{f}^{2}$ remained above 99.9%. As for the shape recovery ratio ($R_{r}$), both $R_{r}^{1}$ and $R_{r}^{2}$ gradually approached 100%. This phenomenon can be interpreted as follows. When fixing the temporary shape one at 80 °C, the shape fixation ratio is primarily determined by the amount of PLLA crystals formed upon cooling to 80 °C. The DSC results in Figure 3 indicate that the crosslinking points significantly suppress the crystallization of PLLA, accounting for the reduced number of crystals in the PLLA SMPs and the consequent decrease in $R_{f}^{1}$. Differently, the shape fixation ratio of the temporary shape two is dependent on the glass transition of PLLA. As a result, the $R_{f}^{2}$ maintains above 99.9%. During the first recovery process, the combination of crystals and crosslinking points synergistically enhanced the shape recovery ratio of PLLA SMPs, leading to the improved $R_{r}^{1}$. Upon heating to 170 °C, the crystals of PLLA melt, transitioning the chains into an amorphous state. In this situation, the crosslinking points play an essential role in determining the shape memory performance. Specifically, the high crosslinking density can effectively minimize chain slippage during the stretching process. Consequently, the $R_{r}^{2}$ exhibits a positive correlation with crosslinking density in these PLLA SMPs.

**Figure 7:**
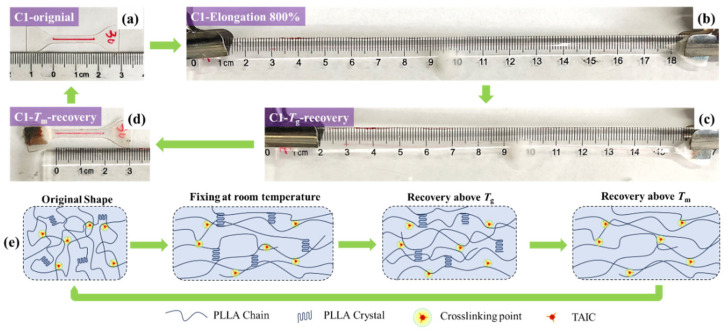
The shape memory process of PLLA SMPs (1 wt% TAIC, 30 kGy): The original form (**a**), strain = 800% at 80 °C (**b**), shape fixing at 80 °C (**c**), and shape recovery at 170 °C (**d**); the schematic illustrations of structure evolution of PLLA SMPs during shape memory process (**e**).

The ultimate recoverable deformation of PLLA SMPs incorporating both crystals and crosslinking points was evaluated and the results are shown in Figure 7. Based on the results of the mechanical property shown in Figure 3d, PLLA SMPs with 1 wt% TAIC were adopted. The specimen was first stretched to a strain of 800% at 80 °C and subsequently fixed in its temporary shape at 25 °C. The shape fixation ratio can maintain 100% at 25 °C. Due to the existence of PLLA crystals in the specimen, the shape fixation ratio can maintain 79.8% upon heating to 80 °C. Heating further to 170 °C, the PLLA crystals melted and the crosslinking points in PLLA SMPs resulted in a recovery behavior with a recovery ratio of 99.3%. The results demonstrated that the crosslinking point endows PLLA SMPs with superior recovery behavior even under extreme deformation conditions.

According to the discussion above, incorporating crosslinking points into PLLA not only introduces additional hard segments, thereby enhancing the recovery stress and shape recovery ratio, but also establishes the melt temperature as a new switching capability. During the dual-shape memory process, both the crosslinking points and the crystals function as hard segments due to their stability above the glass transition temperature (*T*_g_). Furthermore, the crosslinking points in PLLA significantly suppress the cold crystallization of PLLA and prevents the irreversible chain slippage during deformation, contributing to both a high shape recovery ratio and excellent cyclic stability. In the triple-shape memory process, PLLA SMPs with crosslinking points can memorize an original shape and two temporary shapes. The fixation ratio of the first temporary shape decreases due to reduced crystallization during cooling from 170 °C to 80 °C. The other shape memory parameters gradually approach 100% with the development of the crosslinked networks.

## 4. Conclusions

In this work, γ-ray irradiation has been employed to fabricate crosslinked PLLA SMPs based on the miscible blend of PLLA and TAIC. The crosslinking density can be adjusted by the TAIC content and the radiant absorbed dose. The combination of crosslinking points and crystals in crosslinked PLLA SMPs can serve as the hard segments during the shape memory process. Relative to pure PLLA, the crosslinked PLLA exhibits better mechanical property and shape recovery ratio even under extreme deformation conditions (strain = 800%), which can be attributed to the following points. Firstly, the crosslinking point significantly suppresses the cold crystallization during deformation above 80 °C. Secondly, the crosslinking point effectively prevents the irreversible chain slippage during the shape memory process. Both of these contribute to the improved shape recovery ratios (>97.9%) after three cycles. Finally, the stable crosslinking point, together with the crystals, endows PLLA with triple-shape memory performance. Our results provide a novel approach for fabricating high-performance PLLA SMPs with exceptional shape recovery capabilities, even under extreme deformation conditions. These PLLA-based SMPs hold significant promise for biomedical applications, including minimally invasive medical devices, tissue engineering scaffolds, and self-expandable implants. Furthermore, their environmentally benign nature and stimuli-responsive characteristics enable potential use in smart packaging and 4D printing technologies.

## Figures and Tables

**Table 1:** The triple-shape memory performances of PLLA SMPs with different crosslinking densities.

PLLA Specimens	$R_{f}^{1}$ (%)	$R_{f}^{2}$ (%)	$R_{r}^{1}$ (%)	$R_{r}^{2}$
1 wt% 30 kGy	99.9	99.9	94.5	98.4
3 wt% 30 kGy	74.9	99.9	100	99.8
5 wt% 30 kGy	52.7	99.9	100	100
10 wt% 30 kGy	42.2	100	100	100

## Data Availability

Data is contained within the article or Supplementary Material.

## Supplementary Materials

The following supporting information can be downloaded at: https://www.mdpi.com/article/10.3390/polym17223041/s1, Table S1. The mechanical properties of PLLA SMPs at 80 °C; Figure S1. The elasticity modulus of PLLA SMPs at 80 °C; Figure S2. The TGA curves (a) and DTG curves (b) of neat PLLA, PLLA/TAIC blend before and after irradiation.
