# Supplementary material for: Fabrication of Crosslinked Poly(L-lactic acid) with Enhanced Shape Memory Performance via γ-Ray Irradiation
Source: Polymers (Basel). 2025 Nov 17;17(22):3041. doi: 10.3390/polym17223041 (PMC12656042; doi:10.3390/polym17223041)
Supplement: Supplementary file 1 [file polymers-17-03041-s001.zip › polymers-3952472-supplementary.pdf]

Supporting information for

Fabrication of Crosslinked Poly(L-lactic acid) with Enhanced Shape Memory Performance via γ-Ray Irradiation

Jiayao Wang [1,2], Jingxin Zhao [2], Dong Yang [3], Jichun You [2,*] and Guipeng Yu [1,*]

[1] College of Chemistry and Chemical Engineering, Central South University, Changsha 410083, China; jiayaowang@hznu.edu.cn
[2] College of Material, Chemistry and Chemical Engineering, Key Laboratory of Organosilicon Chemistry and Material Technology, Ministry of Education, Zhejiang Key Laboratory of Organosilicon Material Technology, Hangzhou Normal University, Hangzhou 311121, China; adamzhaofu@hotmail.com
[3] Department of Cardiovascular Medicine, Affiliated Hospital of Hangzhou Normal University, Hangzhou 310015, China; xsyd0724@163.com
* Correspondence: you@hznu.edu.cn (J.Y.); gilbertyu@csu.edu.cn (G.Y.)

1. Experiment

Thermogravimetric analysis (TGA) was performed on thermogravimetric analyzer (TA, Q500). The samples were heated at a rate of 10 °C/min from 30°C to 650°C under nitrogen atmosphere. The thermal decomposition temperatures of starting (5% loss) and maximum were evaluated.

2. Figures

Table S1. The mechanical properties of PLLA SMPs at 80 °C

Sample	Elongation at break (%)	Strength at break (MPa)
Neat PLLA	1104±90.5	3.24±0.55
1 %-30 kGy	1086±56.9	12.2±1.87
3 %-30 kGy	750±65.0	24.5±2.09
5 %-30 kGy	580±45.8	21.0±1.76
10 %-30 kGy	432±49.8	18.1±1.43

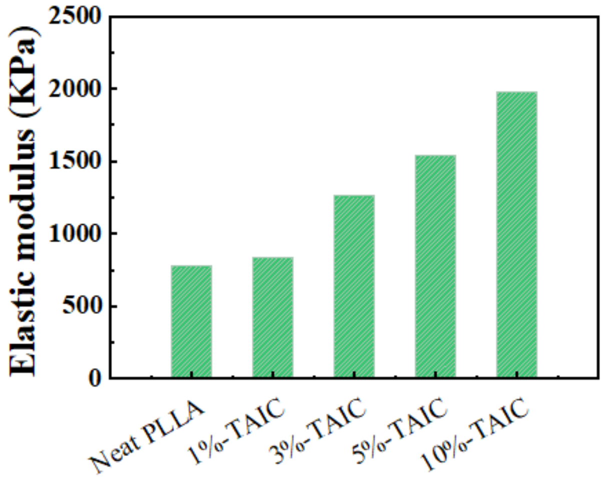

Figure. S1. The elasticity modulus of PLLA SMPs at 80 °C.

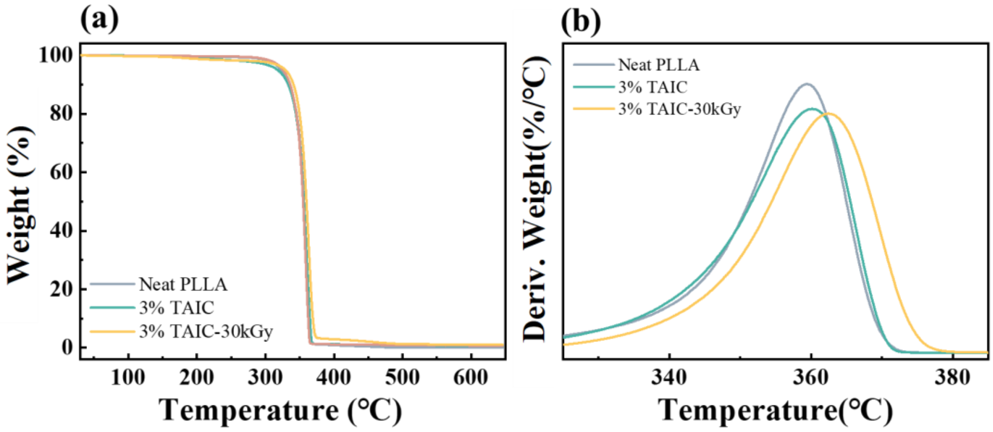

Figure. S2. The TGA curves (a) and DTG curves (b) of neat PLLA, PLLA/TAIC blend before and after irradiation.
